# Integrating single-cell and spatial transcriptomic analysis to unveil heterogeneity in high-grade serous ovarian cancer

**DOI:** 10.3389/fimmu.2024.1420847

**Published:** 2024-06-21

**Authors:** Haixia Luo, Kunyu Wang, Bin Li

**Affiliations:** Department of Gynecological Oncology, National Cancer Center/National Clinical Research Center for Cancer/Cancer Hospital, Chinese Academy of Medical Sciences and Peking Union Medical College, Beijing, China

**Keywords:** HGSOC, spatial transcriptomic, multi-omics, heterogeneity, CHODL

## Abstract

High-grade serous ovarian cancer (HGSOC) presents significant challenges due to its heterogeneity and late-stage diagnoses. Using single-cell and spatial transcriptomics to elucidate the complex landscape of HGSOC to understand its underlying mechanism. Our analysis reveals significant inter- and intra-tumoral diversity, manifested through distinct cellular subpopulations and varied microenvironmental niches. Notably, our findings highlight a widespread immunosuppressive environment, marked by complex networks of cell-cell interactions, particularly evident in areas of elevated tumor cell density within metastatic samples. We identify the exclusive presence of COL14A1+ neoplastic cells in metastatic specimens, alongside a strong correlation between CD8A+ NKT cells and poor prognosis, and elevated CHODL expression in HGSOC metastasis tissues. Furthermore, knockdown experiments targeting CHODL demonstrate its role in reducing migration and invasion abilities in HGSOC cells. A pivotal discovery of our study is the delineation of specific cellular signatures correlated with adverse outcomes, notably a subset of CHODL+ neoplastic cells characterized by a distinct metabolic phenotype with a predilection for lipid metabolism. The therapeutic targeting of this metabolic pathway with existing inhibitors appears promising in curbing tumor proliferation. These findings enhance our understanding of HGSOC heterogeneity and reveal potential therapeutic targets, promising more effective management strategies for this aggressive cancer subtype.

## 1 Introduction

Ovarian cancer ranks as the eighth most common cause of cancer-related deaths among women globally (1). High-grade serous ovarian carcinoma (HGSOC), the deadliest and most prevalent histologic subtype, is responsible for 70–80% of deaths from ovarian cancer (2, 3). It is characterized by an advanced stage at diagnosis and a propensity for rapid metastasis. The genetic architecture of HGSOC is defined by extensive chromosomal instability and signature mutations in key genes such as TP53 and BRCA1/2, alongside numerous disruptions in the pathways responsible for homologous recombination repair (4, 5). These genetic aberrations contribute to the cancer’s diverse clinical manifestations and notable resistance to chemotherapy, underlining the complexity of tumor dynamics and therapeutic outcomes in affected individuals.

The tumor microenvironment (TME), primarily composed of fibroblasts, endothelial cells, lymphocytic infiltrates, and extracellular matrix proteins, can directly influence cancer cell growth, migration, and differentiation, presenting a unique opportunity for diagnosis and treatment (6). The immune system significantly shapes the TME; ongoing inflammation leads to the production of various immunologic gene products that create a favorable microenvironment for tumor growth and progression (7). The presence of specific immune cell types, such as intratumor CD8+ T cells, is associated with improved survival in patients with various cancers, including ovarian cancer (8). These findings indicate that TME heterogeneity especially immune cell, plays a crucial role in determining the malignant phenotypes of cancer cells. However, the heterogeneity of the TME in HGSOC and its association with clinical outcomes, as well as the molecular mechanisms by which various TME components promote or inhibit cancer, remain incompletely understood. Additionally, the interplay between different cellular and non-cellular populations and their spatial organization within the TME in HGSOC requires further elucidation.

Recent advances in single-cell technologies have elucidated the complex cellular landscapes within HGSOC, underscoring the dynamic interactions among cancer cells, immune cells, and stromal elements in the TME. Single cell sequencing enables detailed analysis of transcriptomic characteristics across different cell subsets, revealing cell heterogeneity and microenvironmental features that traditional methods cannot capture (9, 10). However, the aforementioned techniques and analyses cannot provide spatial information. Growing evidence across multiple cancer types indicates that the spatial arrangement of various cellular components within the TME, and their positioning relative to tumor cells, immune cells, and blood vessels, can significantly influence both antitumor and protumor responses (11–13). Spatial transcriptomics technology, capturing genome-wide readouts across biological tissue, enables researchers to determine gene expression spatially within the complex TME (14).

In light of these findings, our study employs scRNA-seq and spatial transcriptomics to dissect the cellular and molecular landscapes of primary HGSOC tumors and their distant metastases. By integrating these state-of-the-art technologies, we aim to uncover the heterogeneity within HGSOC tumors, identify key drivers of metastasis, and elucidate the interactions between tumor cells and the TME that facilitate distant spread. We validated these findings through *in vitro* experiments and immunohistochemistry assays, and corroborated the results using both internal and external data from numerous clinical samples. Our research not only contributes to the understanding of HGSOC biology but also holds promise for identifying novel biomarkers and therapeutic targets to combat HGSOC.

## 2 Materials and methods

### 2.1 Acquisition and processing of bulk transcriptomic data

For this study on ovarian cancer, transcriptomic data, encompassing RNA expression profiles along with relevant clinical details, were sourced from the TCGA database via the “TCGAbiolinks” package (15). To enhance the survival analysis’s reliability, we omitted samples lacking survival data or with a survival duration under 30 days. Subsequently, the data were converted to Transcripts Per Million (TPM) and subjected to log2 transformation in preparation for further analysis (16).

### 2.2 Acquisition and processing of single-cell and spatial transcriptomic data

Single-cell transcriptomic data were meticulously acquired from the GEO database (17), specifically targeting primary tumor samples from GSE211956 and metastatic samples from GSE147082, resulting in a comprehensive collection of 11 samples. The Read10 × function was utilized to process the Seurat object containing the gene expression data of each sample. After quality control of the cells, encompassing data normalization to correct for technical variances, identification of 2,000 highly variable genes to focus on biologically significant fluctuations, and the application of specific functions to mitigate cell cycle effects. Batch effects were corrected using the harmonization technique, ensuring comparability across samples. Dimensionality reduction was achieved through UMAP (Uniform Manifold Approximation and Projection) and t-SNE (t-Distributed Stochastic Neighbor Embedding), revealing inherent data structures, while the Louvain algorithm facilitated insightful clustering, uncovering previously unrecognized cell populations. Differential expression analysis was conducted with stringent criteria, employing a p-value < 0.05, log2 fold change > 0.25, and expression proportion > 0.1, to ascertain significant gene variations across clusters.

In parallel, spatial transcriptomic analysis of 8 primary tumor samples from GSE211956 was executed with precision using “SpaceRanger” for initial quality checks. This was followed by meticulous normalization and variable gene selection via the “SCTtransform” method, optimizing data for subsequent analysis. The spatial data, characterized by an average of 2515 spots per sample, underwent a thorough examination and visualization process through Seurat, enabling a spatially resolved understanding of tumor heterogeneity. The CARD algorithm, informed by single-cell annotations, was applied for cutting-edge spatial data deconvolution, adeptly predicting cell type distributions for each spot, thus bridging the gap between single-cell and spatial transcriptomics. Visualization of spatial cell type distributions and signature score calculations were adeptly achieved using CARD and the “GSVA” package, further complemented by Seurat’s AddModelScore function, providing a multifaceted view of the tumor microenvironment and its dynamic interplay with cancer progression.

### 2.3 Cell annotation

We employed a rigorous and detailed approach to categorize cells within the single-cell transcriptomic datasets. Marker genes were meticulously selected based on established literature and database references to ensure high specificity and sensitivity for each cell type. For epithelial cells, markers included “EPCAM” (epithelial cell adhesion molecule), “KRT18” and “KRT19” (keratins 18 and 19), and “CDH1” (E-cadherin) (18), reflecting their pivotal role in maintaining epithelial integrity and function. Fibroblast identification hinged on the expression of “DCN” (decorin), “THY1” (CD90), “COL1A1”, and “COL1A2”, which are indicative of extracellular matrix production and fibroblast activation (19). Endothelial cells were distinguished by “PECAM1” (CD31), “CLDN5” (claudin-5), “FLT1” (VEGFR-1), and “RAMP2”, markers that denote vascular structures and blood vessel lining (20). T-cells were identified through T-cell receptor components “CD3D”, “CD3E”, “CD3G”, and “TRAC”, underscoring their role in adaptive immunity. NK cell markers “NKG7”, “GNLY” (granulysin), “NCAM1” (CD56), and “KLRD1” (CD94) were chosen for their relevance in innate immune responses. B-cells were annotated using “CD79A”, “IGHM”, “IGHG3”, and “IGHA2”, reflecting their antibody production capabilities. Lastly, mast cells were identified by “KIT” (CD117), “MS4A2” (FcϵRI), and “GATA2”, known for their role in allergic responses and tissue homeostasis. Following cell annotation, we conducted clustering analyses on epithelial cells, immune cells (including T-cells, NK cells, B-cells, and mast cells), and fibroblasts to dissect the tumor heterogeneity further.

### 2.4 Cell culture and lentivirus packaging

The cell lines 293T, ES-2, OVCAR3, SKOV3, OVCA-429, and TOV-21G were procured from the American Type Culture Collection (ATCC; Manassas, VA, USA). A2780 cells were obtained from the National Experimental Cell Resource Sharing Platform (Beijing, China), and 3AO cells were purchased from the Cell Bank of the Chinese Academy of Sciences (Shanghai, China). 293T cells were cultured in DMEM supplemented with 10% fetal bovine serum (FBS), SKOV3 cells in McCoy’s 5A with 10% FBS, and the rest in RPMI 1640 10% FBS.

To generate pLKO.1-puro lentiviruses, HEK293T cells were co-transfected with packaging plasmids psPAX2, pMD2G, and lentiviral vectors. As described previously (21), lentivirus infection was performed in accordance with the manufacturer’s guidelines, and puromycin (2 μg/mL) (540222, Sigma) was applied to establish the stable cellular populations. The CHODL shRNA sequences were as follows (5′-3′): sh1, GCAAGTATGAACCAGAGATTA and sh2, GCATATTCATTGATGAGGGTT.

### 2.5 Tissue microarray

Tissue chips from 125 patients with HGSOC were supplied by the Department of Gynecology Oncology, National Cancer Center/National Clinical Research Center for Cancer/Cancer Hospital. These patients underwent surgical resection from January 2010 to October 2019. Following the exclusion of 3 unsuitable samples, the remaining 119 samples were subjected to further analysis.

### 2.6 Western blotting

Western blotting procedure was performed as described previously (21). Post-blocking with 5% skim milk, the membranes underwent overnight incubation at 4°C with anti-CHODL (diluted at 1:1000; ab236742, Abcam) and anti-GAPDH (diluted at 1:4000; Abclonal) antibodies. Subsequent visualization of the immunoblots was achieved using the ImageQuant LAS-4000 System (GE).

### 2.7 Immunohistochemistry

Tissue microarrays were stained with anti-CHODL antibody (1:100; ab236742, Abcam). The images were captured by Aperio ScanScope (Leica, Nussloch, Germany).

### 2.8 Transwell assays

The migration and invasion capabilities of A2780 cells were evaluated using the transwell assay as described previously (21). Briefly, cells (2 × 10^4^/well) were seeded in 200 μL of serum-free medium. The lower chamber contained 600 μL of medium with 10% FBS. Matrigel (BD Bioscience, USA) was applied to the upper compartment for the invasion assay or omitted for the migration assay. After 24 hours, cells that had invaded or migrated to the lower chamber were stained with 0.1% crystal violet and quantified. Each experiment was conducted independently in triplicate.

### 2.9 Subclustering analysis of cell populations

We adopted a refined approach to discern intricate subpopulations among immune cells, epithelial cells, and fibroblasts, leveraging the robust capabilities of the Seurat package. Initially, we performed a high-resolution clustering analysis, adjusting the resolution parameter in Seurat to detect subtle variations within the broad cell types identified. Differential expression analysis between the identified clusters was then performed to select specific markers for each cell type. The UMAP technique was then employed to visualize the subclusters in a two-dimensional space, allowing for the intuitive interpretation of the complex cellular landscape. To enhance the rigor of our subclustering analysis, statistical tests were incorporated to validate the significance of the identified subclusters.

### 2.10 Copy number variation analysis

We employed the InferCNV software to conduct a comprehensive Copy Number Variation (CNV) analysis on subpopulations of tumor cells, with a specific focus on distinguishing malignant cells within the tumor microenvironment. Immune cells were meticulously chosen as the reference for comparison, based on their stable genomic profile and minimal CNV alterations.

### 2.11 Pseudotime analysis

For the pseudotime analysis within epithelial cell subpopulations, we utilized the Monocle2 software, specifically employing its DDRTree algorithm for effective dimensionality reduction, while adhering to the default settings for other parameters. This analysis was strategically aimed at delineating the cell differentiation trajectory.

### 2.12 Transcription factor analysis

We applied the SCENIC software, maintaining default settings for the RcisTarget and GRNBoost databases. The RcisTarget package was deployed to pinpoint transcription factors notably expressed within our gene list, whereas the AUCell package quantified the activity levels of regulatory networks across identified cell types.

### 2.13 Cell-cell communication

For cell-cell communication assessment, the CellChat package was our tool of choice. Starting with the normalized gene expression matrix, we constructed a CellChat object, followed by the execution of preprocessing functions such as identifyOverExpressedGenes and identifyOverExpressedInteraction, all under default parameters. Subsequent steps involved the computeCommunProb and filterCommunication functions to unveil potential ligand-receptor interactions. The analysis culminated with the aggregateNet function, synthesizing a comprehensive cell communication network.

### 2.14 Statistical analysis

All data processing, statistical analysis, and plotting were performed using R 4.1.3 software. The Pearson correlation coefficient was used to evaluate the correlation between two continuous variables. The chi-square test was employed for comparison of categorical variables, while the Wilcoxon rank-sum test or t-test was used for comparing continuous variables. Cox regression and Kaplan-Meier analysis were conducted using the survival package.

## 3 Results

### 3.1 Single-cell transcriptome atlas of HGSOC

**Figure 1:**
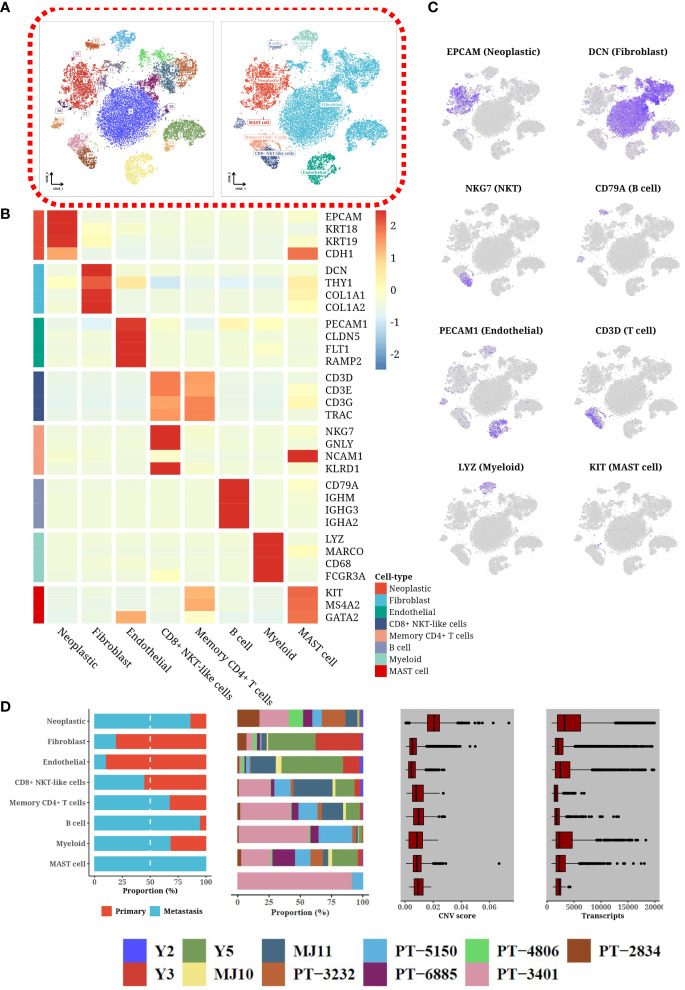
Heterogeneity through single-cell expression profiling. **(A)** tSNE plot categorizing single-cell data by clusters and cell types. **(B)** Heatmap displaying the expression levels of marker genes in different cell types. **(C)** tSNE plot illustrating the expression patterns of various marker genes across different cell types. **(D)** Composite plot showing the composition of cell sources, patient composition, number of cells, number of transcripts detected, and copy number variation (CNV) status for different cell types.

Utilizing single-cell transcriptomics, our investigation revealed a complex cellular landscape within the tumor microenvironment of HGSOC, characterized by the identification of 22 distinct cell clusters. This diversity was elucidated through the detection of specific markers, such as EPCAM and KRT for epithelial cells, DCN and COL1A1 for fibroblasts, PECAM1 and CLDN5 for endothelial cells, and various others for immune cell types including T-cells, NK-cells, B-cells, and mast cells (**Figure 1A**). Sophisticated visualization methods, like heatmaps (**Figure 1B**) and t-SNE plots (**Figure 1C**), effectively demonstrated the unique expression patterns of these markers, highlighting the considerable heterogeneity among the cells present. The deployment of scType software further refined the classification of immune cells, providing deeper insights into the intricacies of the tumor’s cellular composition. Notably, these cell types were present in nearly all patients, albeit in differing proportions (**Figure 1D**). Tumor cells predominated, exhibiting elevated levels of transcripts and copy number variations, indicative of their malignant nature. Among immune cells, T-cells were most prevalent, with a notable reduction in B cells, myeloid cells, and mast cells in primary tumors compared to metastatic samples. Conversely, stromal cells were significantly diminished in the tumor microenvironment of metastatic samples, likely due to the expansion of tumor cells.

### 3.2 Heterogeneity through spatial transcriptomic expression profiling

**Figure 2:**
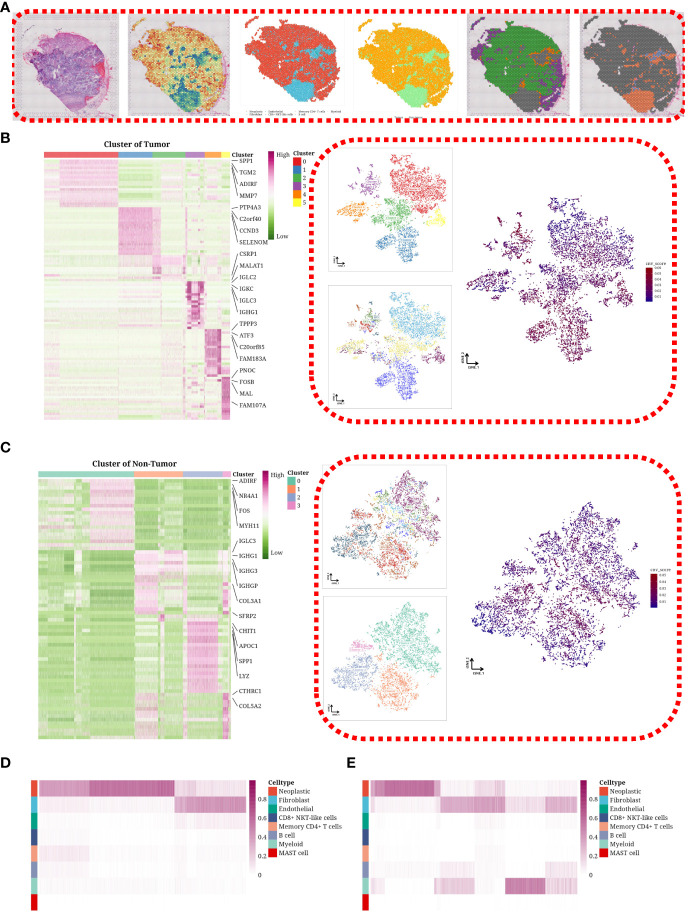
Heterogeneity through spatial transcriptomic expression profiling. **(A)** H&E staining image, distribution of CNV scores, cellular composition of the tumor sample, tumor or non-tumor classification, clustering results of tumor cells, and clustering results of non-tumor cells in representative SP2 sample. **(B)** Heatmap showing marker expression of tumor cell subgroups on the left, and tSNE plot illustrating the composition, CNV score, and patient distribution of tumor cell subgroups on the right. **(C)** Heatmap showing marker expression of non-tumor cell subgroups on the left, and tSNE plot illustrating the composition, CNV score, and patient distribution of non-tumor cell subgroups on the right. **(D)** Heatmap depicting the cellular composition differences in poor_response samples. **(E)** Heatmap illustrating the cellular composition differences in good_response/partial_response samples.

We applied the inferCNV software to evaluate copy number variations (CNVs) across eight designated spots within each sample. To dissect the cellular composition of the spatial transcriptomic data, we implemented the CARD algorithm. Spots explicitly identified as tumor cells were categorized accordingly, with remaining cells labeled as non-tumor. For a representative sample (SP1), we showcased a range of visualizations, including hematoxylin and eosin (H&E) staining, CNV score distribution, cellular composition, and clustering results for both tumor and non-tumor cells (**Figure 2A**). SP1, SP4, SP7, and SP8, linked to suboptimal or partial chemotherapy responses, exhibited a predominant tumor cell presence (data not displayed). Further analysis involved segregating spots into tumor and non-tumor groups, followed by dimensional reduction and clustering. The tumor cells were classified into six subgroups, with each subgroup’s composition, CNV status, and marker genes elucidated on a tSNE plot (**Figure 2B**). Non-tumor cells were divided into four subgroups (**Figure 2C**). A comparative heatmap analysis between samples showing poor responses and those with better or partial responses highlighted a greater prevalence of tumor cells in the former and an increased presence of non-tumor cells, such as fibroblasts, in the latter (**Figures 2D, E**).

### 3.3 High heterogeneity of neoplastic cells

**Figure 3:**
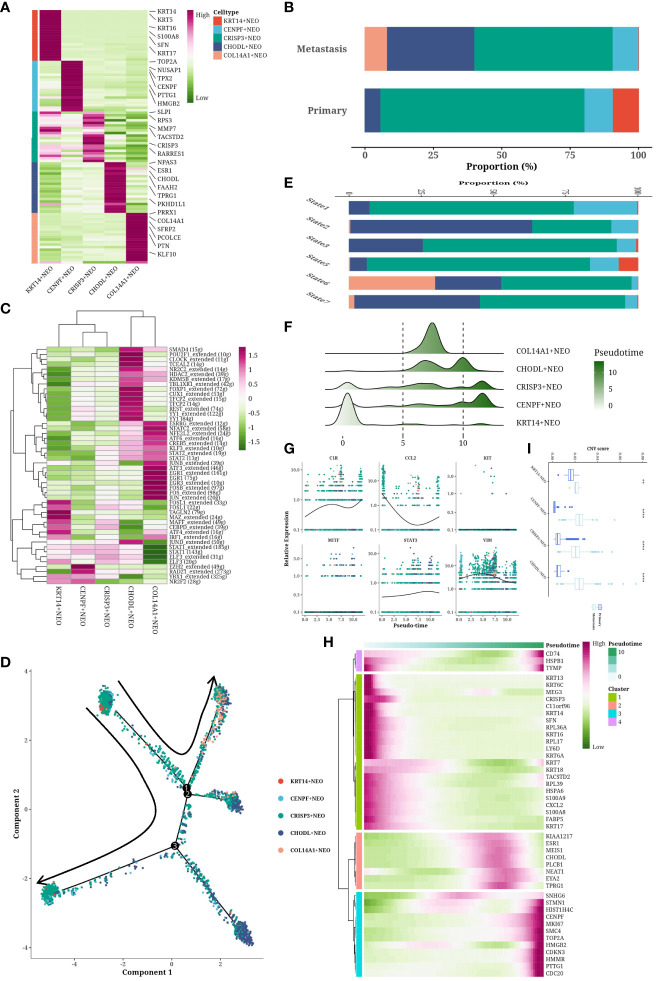
Subtyping analysis of tumor cells. **(A)** Cell communication network map of two cell groups. **(B, C)** Cell communication scatterplot depicting intercellular interactions between two cell groups. **(D)** Chord diagram illustrating the signaling pathways of MK. **(E)** Chord diagram depicting the signaling pathways of GALECTIN. **(F)** Spatial expression maps showing the distribution of LGALS9_CD44 in representative samples. **(G)** Spatial expression maps showing the distribution of LGALS9_HAVCR2 in representative samples.

Neoplastic (NEO) cells were segregated and subjected to dimensionality reduction and clustering, unveiling five unique subgroups (**Figure 3A**). A bar graph delineated the cell composition from varied origins, highlighting KRT14^+^ NEO cells in primary samples and COL14A1**+** NEO cells in metastatic counterparts (**Figure 3B**). Transcription factor (TF) profiles for each subgroup were analyzed, with a heatmap illustrating the enrichment of specific TFs, such as the heightened activity of FOSL1 in KRT14+ NEO cells indicative of an immunomodulatory phenotype. FOSB and JUNB were notably active in COL14A1^+^ NEO cells, implicating their roles in differentiation, proliferation, and apoptosis (**Figure 3C**). Cellular trajectory analysis, using primary cells as a reference, depicted multiple developmental pathways within metastatic samples, indicating subtype diversity (**Figure 3D**). These pathways culminated in branches rich in CHODL^+^ NEO cells. Pseudotemporal analysis positioned KRT14^+^ NEO cells at the onset, whereas COL14A1^+^ NEO cells were intermediate, with the bulk of cells at the trajectory’s end (**Figures 3E, F**). A scatter plot illustrated the pseudotemporal evolution of six genes (MITF, KIT, VIM, CCL2, C1R, STAT3), with a heatmap showcasing gene expressions linked to pseudotemporal progression (**Figures 3G, H**). This analysis revealed a loss of the immune-related molecule CCL2 over time, contributing to a “cold” tumor microenvironment, and an increase in metastasis-associated molecules like MITF, KIT, and VIM, suggesting a potential transition to a mesenchymal phenotype. CNVs were distinct across cell types and origins (**Figure 3I**). Metastatic NEO cells, especially those expressing COL14A1, not analyzed in primary samples due to their exclusivity to metastatic sites, displayed a more malignant profile than their primary counterparts, with CHODL^+^ NEO cells at the developmental culmination exhibiting the highest CNV levels.

### 3.4 Functional analysis of endpoint cells (CHODL^+^ NEO cells)

In our comparative analysis of primary and metastatic tumor samples, we noted a markedly higher prevalence of CHODL^+^ NEO cells in metastatic specimens (**Supplementary Figure S1A**). A heatmap delineating gene expression differences between CHODL^+^ NEO cells and their counterparts underscored the upregulation of genes associated with lipid metabolism in the former group (**Supplementary Figure S1B**). We further explored lipid metabolism pathways, computing signature scores for both cell populations and illustrating the disparities through heatmap visualization, which highlighted distinct variations in the FATTY_ACID_BETA_OXIDATION pathway (**Supplementary Figure S1C**). Additionally, SMAD4 was identified as a significantly enriched transcription factor in CHODL+ NEO cells, with its expression profile analyzed within a control dataset. We examined the distribution of FATTY_ACID_BETA_OXIDATION pathway scores in patients SP1 and SP6, categorizing cells based on SMAD4 expression with the mean as a threshold (**Supplementary Figure S1D**). Violin plots underscored significant disparities in pathway scores between the two groups (**Supplementary Figures S1E, F**), underscoring the unique metabolic phenotype of CHODL^+^ NEO cells.

### 3.5 The anti-tumor immune function of metastatic samples was impaired

**Figure 4:**
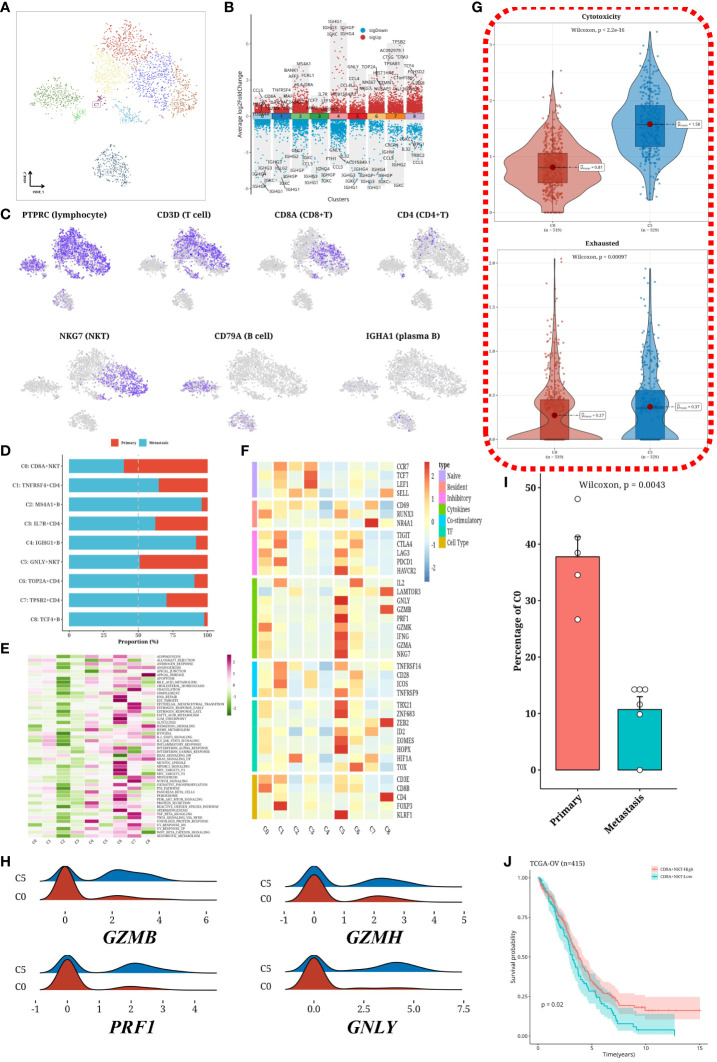
Subtyping analysis of T/B cells. **(A)** tSNE plot showing subgroups of T cells and B cells. **(B)** Volcano plot depicting differentially expressed genes in each subgroup. **(C)** tSNE plot illustrating the expression of immune cell markers. **(D)** Bar graph displaying the distribution of T cell and B cell subgroups across different samples. **(E)** Heatmap showing the scores of Hallmark gene sets in each subgroup. **(F)** Heatmap demonstrating the expression of immune-related genes in each subgroup. **(G)** Violin plot comparing the Cytotoxicity and Exhausted values of different subgroups of CD8+ T cells. **(H)** Ridge plot showing the expression levels of four immune-related genes (GZMB, GZMH, PRF1, GNLY) in CD8+ T cell subgroups. **(I)** Bar graph representing the proportion of C0 subgroup in primary and metastatic samples. **(J)** Survival analysis results of C0 (CD8A+NKT) cell content in the TCGA-OV cohort.

T and B cells were isolated from the sample and subjected to dimensionality reduction clustering analysis, which identified nine distinct cell clusters (**Figure 4A**). Differential gene expression analysis was then performed for each cluster, visually represented as a volcano plot (**Figure 4B**). To validate cell subtypes, t-SNE plots were utilized to illustrate the expression patterns of subtype-specific marker genes (**Figure 4C**). The distribution of cells within each cluster was graphically depicted using a bar chart (**Figure 4D**). Notably, metastatic samples exhibited a significant decrease in C0 (CD8A^+^ NKT) cells and a significant increase in most B-cells and CD4+ T cells compared to primary samples. Hallmark gene set scores were computed for each cluster and visualized in another heatmap (**Figure 4E**), indicating stronger activation levels in C6 and C7. Furthermore, immune-related gene expression profiles across different clusters were elucidated through an additional heatmap (**Figure 4F**). In metastatic samples, there was a notable increase in cells with immunosuppressive effects and a significant loss of cells with cytotoxic functions. To further assess CD8^+^ cells, cytotoxicity and exhaustion values were determined, revealing notable differences among different CD8^+^ cell subgroups (**Figure 4G**). Additionally, the expression distributions of four immune-related genes (GZMB, GZMH, PRF1, GNLY) within CD8^+^ cells (C0 and C5) were visualized (**Figure 4H**). Comparing the proportions of C0 in primary and metastatic samples, a significantly higher content was observed in the primary samples (**Figure 4I**). Importantly, our analysis unveiled a correlation between the diminished abundance of C0 (CD8A^+^ NKT) cells and poorer survival outcomes in the TCGA-OV cohort (**Figure 4J**).

### 3.6 Decreased CCL3-secreting M1 macrophages accelerate metastasis

**Figure 5:**
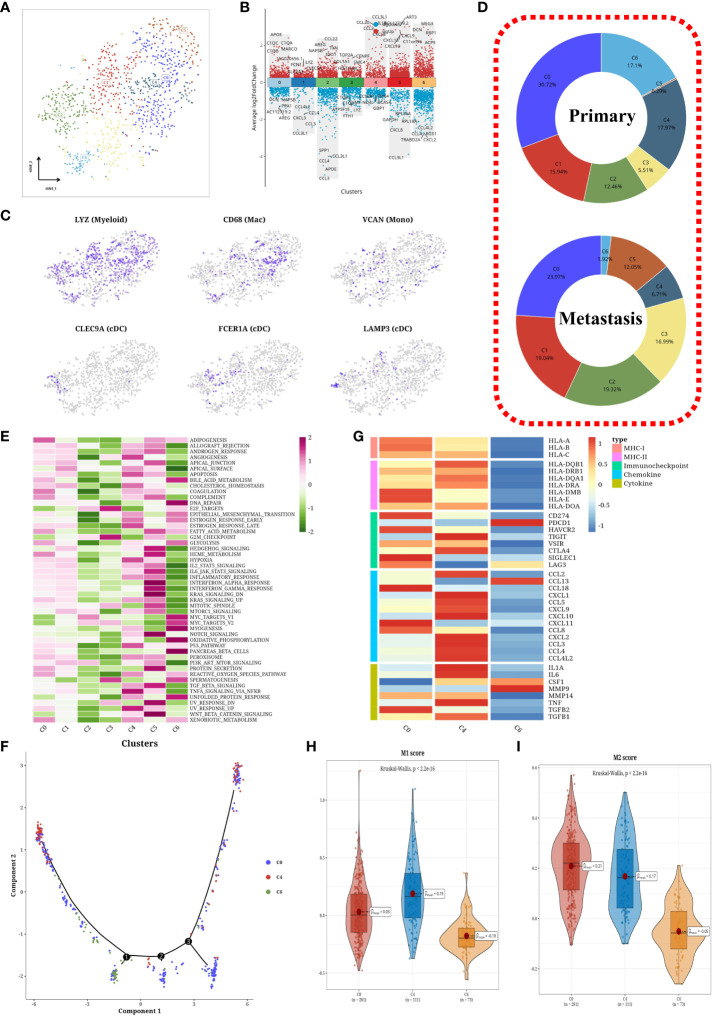
Subtyping analysis of myeloid cells. **(A)** tSNE plot of subpopulations of myeloid cells. **(B)** Volcano plots showing differentially expressed genes among each subpopulation. **(C)** tSNE plot of marker expression levels in myeloid cells. **(D)** Pie charts depicting the composition of subpopulations in primary and metastatic samples. **(E)** Heatmaps displaying scores of hallmark gene sets in each subpopulation. **(F)** Cell trajectory analysis plot of macrophages. **(G)** Heatmaps displaying expression levels of immune-related genes in macrophage subpopulations. **(H)** Violin plots showing differential M1 score among subpopulations of macrophages. **(I)** Violin plots showing differential M2 score among subpopulations of macrophages.

Through isolation and dimensionality reduction clustering, we delineated seven myeloid cell subtypes, illustrated in **Figure 5A**. A volcano plot highlighted differentially expressed genes across these subgroups (**Figure 5B**). t-SNE visualizations confirmed our classifications, showcasing gene expression through subtype-specific markers (**Figure 5C**). A pie chart detailed the composition of primary versus metastatic subgroups (**Figure 5D**), revealing notable shifts: a decrease in C4 (CCL3^+^) cells, and an increase in C2 (CCL22^+^), C3 (TOP2A^+^), and C5 (ART3^+^) within metastatic samples. Hallmark pathway activation levels were analyzed, with C4 and C5 showing heightened activity (**Figure 5E**). Macrophage trajectory analysis from primary cells (C0+C4+C6) unveiled a unique differentiation pathway (**Figure 5F**), and a heatmap of immune gene expression within macrophage clusters underscored an active immune function in C4 (**Figure 5G**). Violin plots further elucidated significant differences between subgroups (**Figures 5H, I**), suggesting C4 as pro-inflammatory M1 macrophages, C0 as M2 macrophages, and C6 as undifferentiated M0 macrophages.

### 3.7 Subtyping analysis of stroma cells

**Figure 6:**
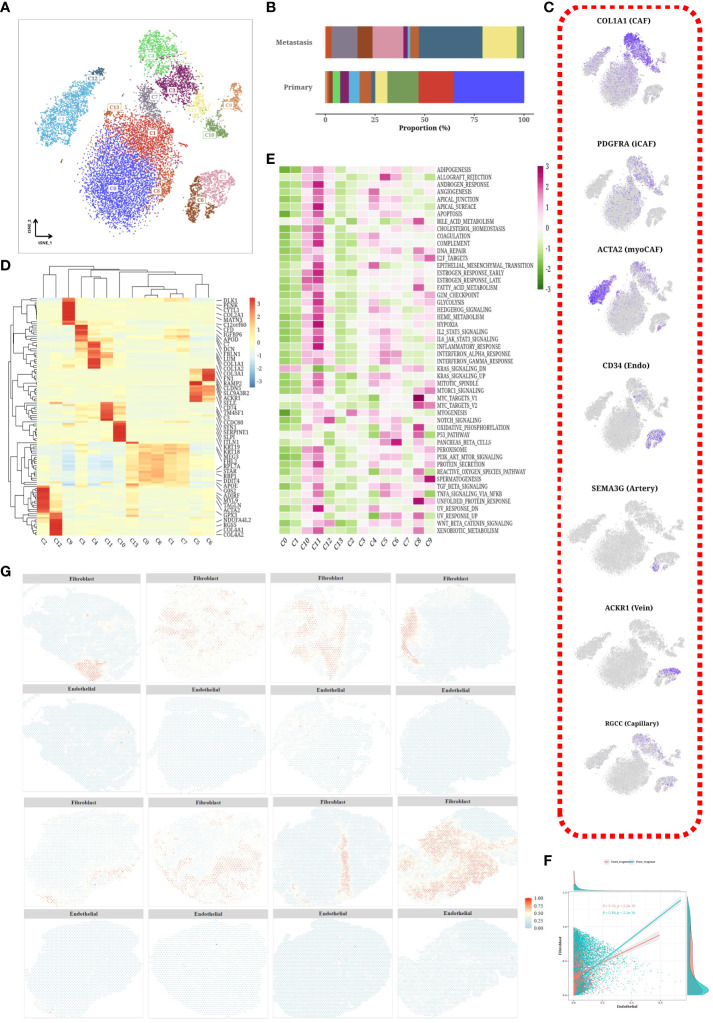
Subtyping analysis of stroma cells. **(A)** tSNE plot of subpopulations of stromal cells. **(B)** Bar chart showing the proportion of each subpopulation in different samples. **(C)** tSNE plot of marker expression levels in stromal cell subpopulations. **(D)** Heatmap displaying differentially expressed genes among each subpopulation. **(E)** Heatmap displaying scores of hallmark gene sets in each subpopulation. **(F)** Scatterplot showing correlation between content of endothelial cells and fibroblasts. **(G)** Spatial distribution of content of fibroblasts and endothelial cells in different tumor samples.

We isolated stromal cells and subjected them to dimensionality reduction clustering analysis, which enabled us to identify 14 different cell subtypes and determine their corresponding cellular origins (**Figures 6A, B**). To ensure the accuracy of our findings, we employed marker genes specific to each subtype and visualized their expression patterns using t-SNE plots (**Figure 6C**). Notably, we observed that myoCAF, iCAF, and endothelial cells were clustered separately. Subsequently, we performed differential gene expression analysis within each subgroup and presented the results in a differential heatmap (**Figure 6D**). To gain further insight into the characteristics of each subgroup, we evaluated the Hallmark gene set scores and generated a corresponding heatmap (**Figure 6E**). Interestingly there was a positive correlation between endothelial and fibroblast expression (**Figure 6F**). Moreover, we investigated the abundance of fibroblasts and endothelial cells across the spatial transcriptomic samples and found that the content of fibroblasts was higher than that of endothelial cells (**Figure 6G**).

### 3.8 Cell communication analysis

**Figure 7:**
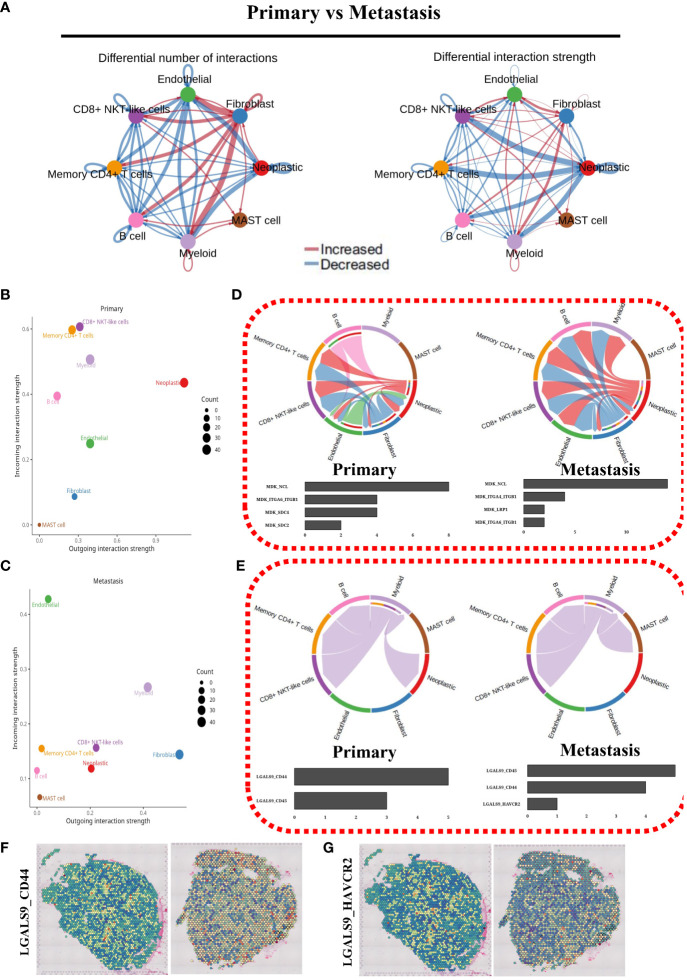
Cell communication analysis. **(A)** Cell communication network map of two cell groups. **(B)** Cell communication scatterplot depicting intercellular interactions between two cell groups. **(C)** Chord diagram illustrating the signaling pathways of MK. **(D)** Chord diagram depicting the signaling pathways of GALECTIN. **(E)** Spatial expression maps showing the distribution of LGALS9_CD44 in representative samples. **(F)** Spatial expression maps showing the distribution of LGALS9_HAVCR2 in representative samples.

We conducted an in-depth analysis of cellular communication in both primary and metastatic cells. This analysis allowed for a comprehensive comparison of the results, enabling us to generate informative network graphs (**Figure 7A**) and scatter plots (**Figures 7B, C**) that visually depict the intricate patterns of cellular communication. To further investigate this phenomenon, we carefully examined the chord diagrams representing the MK and GALECTIN signal pathways. As a result, we constructed compelling statistical bar charts (**Figures 7D, E**) that effectively illustrate the ligand-dependent receptor signaling dynamics. Additionally, we quantified the expression of LGALS9_CD44 and LGALS9_HAVCR2 (**Figures 7F, G**) in spatial transcriptomic samples. Notably, our findings clearly demonstrated a significantly higher expression level of LGALS9_CD44 compared to LGALS9_HAVCR2.

### 3.9 Experimental validation of CHODL

**Figure 8:**
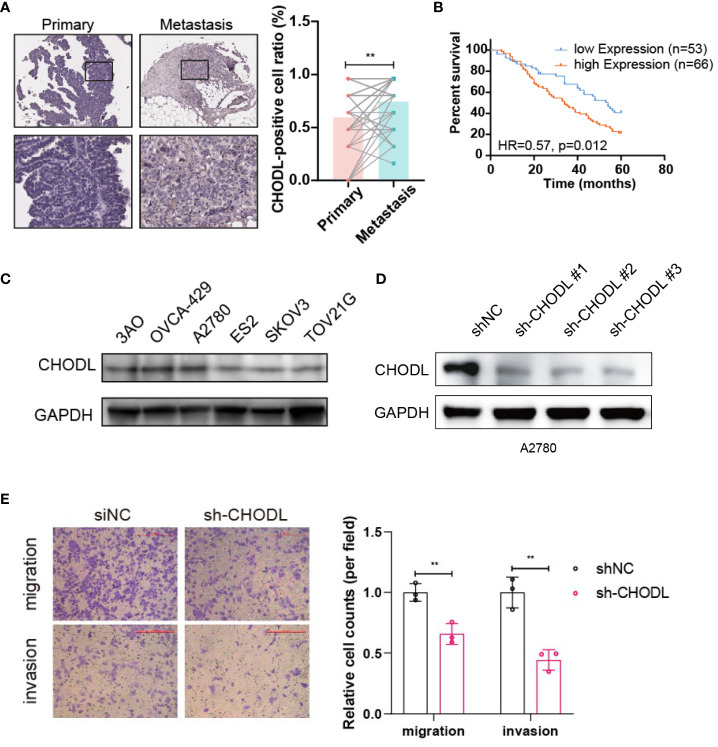
CHODL regulates migration and invasion in HGSOC cells. **(A)** Immunohistochemical analysis of CHODL expression in 52 paired samples from primary and metastatic HGSOC tissues. **(B)** The survival data analysis of 119 HGSOC patients. **(C)** Immunoblot depicting the expression of CHODL protein in 6 common HGSOC cell lines. **(D)** Immunoblot demonstrating CHODL expression levels in A2780 cells following CHODL gene knockdown. **(E)** Transwell assays evaluating the effects of CHODL on the invasion capabilities of A2780 cells. Scale bar represents 200 μm. Statistical analysis of transwell assays is presented in the right. The data are presented as the mean ± s.d. values; t-test, **p < 0.01; n = 3.

Given the higher risk coefficient of CHODL, we examined the tumor tissues from HGSOC patients, and found that the expression of CHODL was higher in the metastasis tissues compared to that of primary tissues (**Figure 8A**). Furthermore, high expression of CHODL was associated with worse prognosis (**Figure 8B**). We selected CHODL for the experimental validation. As shown in **Figure 8C**, CHODL protein expression was relatively high in the A2780 cell line. Therefore, we knocked down the gene in A2780 cell line (**Figure 8D**) to further determine its biological significance. Transwell assays demonstrated that knockdown of CHODL strongly inhibited cell migration and invasion in A2780 cells (**Figure 8E**).

## 4 Discussion

80% of HGSOC patients present abdominal metastasis initially, which is a foothold for subsequent tumor recurrence and unfavorable prognosis. In this comprehensive investigation, we meticulously analyze the intricate landscape of HGSOC using single-cell and spatial transcriptomic analyses. We aim to discern the transcriptome landscape of the tumor/immune interactions in primary or metastatic HGSOC tissues across spatial and temporal dimensions. Our findings reveal pronounced inter- and intra-tumor heterogeneity in HGSOC, accompanied by a highly suppressive TME and intricate cell-cell communications, particularly in metastatic HGSOC. Notably, CHODL+NEO and CD8A+ NKT cells correlate closely with metastasis and poorer prognosis among HGSOC patients. Further clinical analysis and experiments support CHODL’s potential role as a pivotal regulatory factor in HGSOC metastasis, making it a promising therapeutic target.

We highlighted the considerable intratumoral and intertumoral variability in HGSOC, presenting formidable challenges in the realms of treatment efficacy and prognostication. Through the identification of distinct cellular populations at both primary and metastatic sites, our study elucidates the evolutionary trajectory of tumor cells, shedding light on the metastatic cascade. This diversity spans not only the tumor cells but also the stromal and immune constituents within the TME, all contributing to the tumor’s adaptive resilience. Notably, the identification of specific subpopulations, such as CHODL^+^ NEO cells, associated with adverse outcomes and a propensity for metastasis, provides critical insights into the cellular mechanisms underpinning HGSOC dissemination. This concept of cell subclusters driving metastasis resonates with findings in melanoma, where specific cell states are linked to tumor advancement (22, 23). Chondrolectin (CHODL) has emerged as a significant molecular player in the realm of cancer research, particularly in colorectal cancer (CRC) (24) and non-small cell lung cancer (NSCLC) (25). The aberrant hypermethylation of CpG islands is a hallmark of CRC, and CHODL has been identified as a novel gene preferentially methylated in human CRC. Notably, the downregulation of CHODL in CRC, driven by promoter hypermethylation, has been associated with poor survival rates, especially in patients with early-stage CRC (24). Moreover, the expression of CHODL, screened through a comprehensive analysis of gene transactivation in lung cancers, has been correlated with the clinicopathologic significance in patient tissues. The strong positivity of CHODL protein is linked with shorter survival rates in NSCLC patients, underscoring its potential as an independent prognostic factor (25). Given the relatively high-risk coefficient of CHODL in the model, we further validated this gene in cellular models of HGSOC. Our findings suggest that CHODL is a metastasis-related gene that promotes HGSOC cell migration and invasion, and its high expression in the tumor tissues is associated with poor prognosis. However, the mechanism and the participated signaling pathway of CHODL in HGSOC remains to be further investigated, and the possibility of CHODL as a therapeutic target will be further explored in our subsequent study.

We further elucidate the tumor’s metastatic potential through the lens of cellular pathways exploitation, notably those regulated by SMAD4. The augmented activation of lipid metabolism pathways, potentially mediated by SMAD4 in CHODL^+^ NEO cells, underscores the complexity of cancer progression mechanisms. SMAD4’s role as a central mediator in the TGF-β signaling pathway, regulating key cellular processes such as proliferation, differentiation, and apoptosis, is well-documented, with its involvement in cancer highlighting its potential as a therapeutic target. SMAD4, a central mediator in the TGF-β signaling pathway (26), plays a pivotal role in the regulation of cellular processes such as proliferation, differentiation, and apoptosis (27). SMAD4’s presence was found to impart vulnerability to ferroptosis, a form of regulated cell death, in highly invasive tumor cells induced by TGF-β1 (28). Ferroptosis is distinct from other forms of cell death, such as apoptosis, and is characterized by the accumulation of iron-dependent lipid peroxides (29). The implication of SMAD4 in ferroptosis suggests a nuanced role in cancer cell survival and death, offering potential therapeutic avenues to exploit this vulnerability in SMAD4-positive cancers.

The highly immunosuppressive TME observed in HGSOC, particularly in tumors with distant metastases, underscores the challenges in harnessing the immune system for therapeutic benefit. The increase in exhausted cells, alongside a decrease in CD8A^+^ NKT cells in tumors with metastatic involvement, paints a picture of a TME adept at evading immune surveillance. The decrease in CD8A^+^ NKT cells, known for their potent anti-tumor activity (30), in metastatic tumors highlights a potential mechanism by which HGSOC evades immune-mediated destruction. This finding is particularly intriguing given the emerging role of NKT cells in cancer immunotherapy (31). Research has shown that CD8A^+^ NKT-like cells not only possess cytotoxic granules, indicative of their potential to directly engage and destroy tumor cells, but also secrete high levels of interferon-gamma (IFN-γ) when stimulated by TCR-matched antigens (32). The secretion of IFN-γ is particularly noteworthy as it plays a crucial role in antitumor immunity by activating other immune cells and increasing the immunogenicity of cancer cells. Further insights into the distinct populations and functional specializations of NKT cells, including CD8A^+^ NKT-like cells, highlight the complexity of the immune system’s interaction with cancer (33). These cells’ unconventional lifestyles and roles in tumor immunity underscore the potential for novel therapeutic strategies that harness their unique capabilities.

The complex cell-cell communications unveiled in our study, especially pronounced in tumors with distant metastases, reveal a sophisticated network of interactions that facilitate tumor progression. The dialogues between stromal, tumor, and immune cells underscore the collaborative nature of the TME in promoting tumor growth and evasion from immune surveillance. This complexity is further exemplified by the role of metabolic reprogramming, particularly the shift towards lipid metabolism in facilitating metastasis. One of the key findings in this area is the discovery of various mechanisms through which lipid metabolism promotes tumor growth and survival, many of which operate independently of traditional cellular bioenergetics. For example, the reprogramming of lipid metabolism in cancer cells can lead to the accumulation of specific lipid species that contribute to the malignant phenotype, influencing processes such as ferroptotic-mediated cell death, tumor metastasis, and interactions with immune cells within the tumor microenvironment (34). Lipid droplets, in particular, have been identified as crucial players in cancer, acting as reservoirs for energy storage and sources of signaling molecules that can protect cancer cells under stressful conditions, such as hypoxia or nutrient deprivation (35). The therapeutic potential of targeting the lipid metabolism pathway, as demonstrated by the efficacy of etomoxir in suppressing metastasis, opens new avenues for intervention (36). This approach aligns with the growing interest in targeting metabolic pathways as a means to thwart cancer progression.

Utilizing single-cell sequencing technology, we have been able to thoroughly analyze the transcriptomic characteristics of different cell subsets in tumor tissues. single-cell sequencing has enabled us to deeply explore cellular heterogeneity and microenvironment characteristics, identify rare cell subpopulations, examine cell-cell interactions, and uncover potential therapeutic targets that traditional methods could not achieve. New sequencing techniques with spatial resolution deepen our understanding of the relationship between a cell’s genotype or gene expression and its morphology and interactions with the local environment, thus advancing knowledge in HGSOC development and progression.

While our study provides significant insights into the cellular and molecular underpinnings of HGSOC metastasis, several limitations warrant consideration. The heterogeneity among patients and the limited sample size pose challenges in generalizing our findings. The dynamic nature of cancer biology means that our findings are only a snapshot of time, which may not fully cover the complexity of tumor heterogeneity and the dynamics of TME. Addressing the limitations noted, future studies should aim to include larger and more diverse cohorts. This expansion would allow for a broader generalization of our findings and enable a more detailed validation of the observed phenomena. Additionally, investigating the common mechanisms of metastasis in HGSOC could significantly enhance our understanding of the disease process and potentially unveil novel therapeutic targets. Moreover, advancing beyond the two-dimensional spatial analyses employed in our current study, future research should incorporate three-dimensional imaging techniques. Such approaches will provide a more comprehensive view of the tumor architecture, improving our understanding of how the three-dimensional environment influences cellular interactions and tumor progression. This could lead to breakthroughs in how we approach the prevention and intervention of metastasis in HGSOC.

In conclusion, our study sheds light on the intricate tapestry of cellular heterogeneity, immune evasion, and metabolic reprogramming in HGSOC, particularly in the context of distant metastasis. By unraveling the complex interplay between tumor cells and their microenvironment, we pave the way for novel therapeutic strategies aimed at disrupting these interactions.

## Data availability statement

The original contributions presented in the study are included in the article/**Supplementary Material**. Further inquiries can be directed to the corresponding author.

## Ethics statement

Ethical approval was not required for the studies on humans in accordance with the local legislation and institutional requirements because only commercially available established cell lines were used.

## Author contributions

HL: Investigation, Validation, Writing – original draft. KW: Investigation, Methodology, Software, Writing – original draft. BL: Funding acquisition, Supervision, Writing – review & editing.

## Supplementary material

Supplementary Figure 1 Functional Analysis of CHODL+NEO. **(A)** Bar graph depicting the proportion of CHODL+NEO cells in different sample sources. **(B)** Heatmap showing the differential gene expression between CHODL+NEO cells and non-CHODL+NEO cells. **(C)** Heatmap displaying the signature scores of lipid metabolism-related pathways in CHODL+NEO cells and non-CHODL+NEO cells. **(D)** Representative spatial expression maps illustrating the scores of SMAD4 and FATTY ACID BETA OXIDATION. **(E)** Violin plots demonstrating the differences in FATTY ACID BETA OXIDATION scores in SP1. **(F)** Violin plots demonstrating the differences in FATTY ACID BETA OXIDATION scores in SP6.
