# Supplementary figures and images for: Integrating single-cell and spatial transcriptomic analysis to unveil heterogeneity in high-grade serous ovarian cancer
Source: Front Immunol. 2024 Jun 21;15:1420847. doi: 10.3389/fimmu.2024.1420847 (PMC11224428; doi:10.3389/fimmu.2024.1420847)

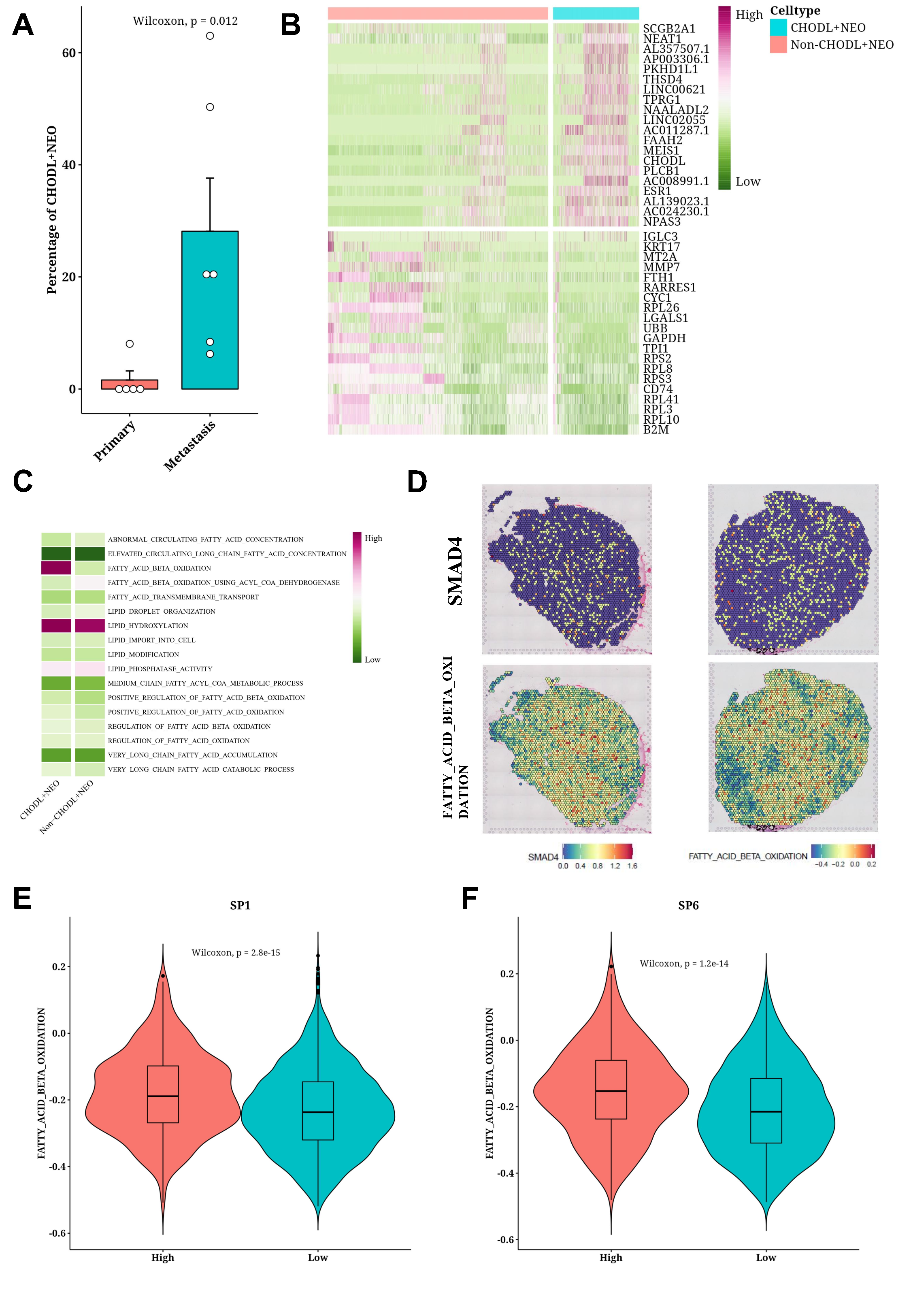

Supplement: Supplementary Figure 1 — Functional Analysis of CHODL+NEO. (A) Bar graph depicting the proportion of CHODL+NEO cells in different sample sources. (B) Heatmap showing the differential gene expression between CHODL+NEO cells and non-CHODL+NEO cells. (C) Heatmap displaying the signature scores of lipid metabolism-related pathways in CHODL+NEO cells and non-CHODL+NEO cells. (D) Representative spatial expression maps illustrating the scores of SMAD4 and FATTY ACID BETA OXIDATION. (E) Violin plots demonstrating the differences in FATTY ACID BETA OXIDATION scores in SP1. (F) Violin plots demonstrating the differences in FATTY ACID BETA OXIDATION scores in SP6. [file Image_1.tif]
